# Personalisation of treatment pathways – analysis of chances and barriers by the implementation of digital technologies under the conditions of the German Health System

**DOI:** 10.12688/f1000research.29969.1

**Published:** 2021-02-25

**Authors:** Armin Töpfer, Georg Brabänder

**Affiliations:** 1 TU Dresden, Faculty of Business and Economics, Research Group Corporate Management and Marketing, Dresden, 01069, Germany

**Keywords:** Personalisation of treatment pathways, Health Services 4.0, cyber-physical systems, translation of innovations, customer journey, ambidexterity

## Abstract

**Background:** The potential of digital technologies is far from being exhausted for patients. The regulatory framework becomes a brake on innovation due to digitalisation, but also due to the trend towards individualisation. Strategies, corporate culture and processes, which are necessary for the design of high-quality and cost-effective healthcare services, are still lacking in many healthcare providing organisations.

**Health Services 4.0 and patient integration as leverage:** With Health Services 4.0 it is possible to improve the outcome of the individual healthcare service and meet the regulatory requirements. This requires the capabilities of the provider to dynamically balance exploitation and exploration. The challenges are to develop innovations in a continuously changing working environment and/or to adapt (medical) technical innovations into their own service processes.

**Conclusion:** This article is focused on hypotheses of cause-and-effect analyses formulated as scenarios, related to the implementation of digital technologies in order to improve efficiency and effectiveness for a high medical expertise as well as for a higher level of service quality.

The output is a more detailed analysis of key value drivers, success factors as well as internal and external value generators for the design of Health Services 4.0. Up to now many issues regarding the use of digital technologies are still only partly analysed and not yet proved for a more efficient care on high-quality level.

The company's capacity for ambidexterity is becoming an important dynamic capability, with on one hand flexibility for new developments and on the other hand stability for hard factors in physical value chains and soft factors in value-oriented attitudes and behaviour based on empathy.

This article was previously published in German in "Monitor Versorgungsforschung" under the original title " Personalisierung von Behandlungspfaden – Das Potenzial digitaler Technologien". This translated version faithfully reflects the authors, data, and interpretations of the original.

## Introduction

The German Healthcare system shows a wide variety of specific features compared to other healthcare systems. Examples include the delegation of supply contracts to associations of service providers, while simultaneously regulating the content of service, as well as the third payer principle of financing. Thus, suppliers at the provider level see themselves confronted with a multitude of legal regulations concerning the integration of the patient with joint decision-making, data protection and quality assurance. Simultaneously, there exists demand for a more economic provision of services as well as high- quality medical care with rapid availability. Technological innovations, such as big data analytics applications, sensor technology and the Internet of Things, analyses of OMICS technologies, augmented reality and robotics have the potential to change the previously existing – largely analogue – value chains through integration of the patient with the combination of conventional healthcare with digital technologies, or Healthcare Services 4.0. The patient’s needs are tailored to individual needs in the form of personalised treatment pathways, increasing the effectiveness and the efficiency of health services.

Existing literature focuses mainly on individual applications of digital technologies in regard to specific disease patterns. Increasing digitalisation is accompanied by a change in work and organisational structures as well as a change in leadership culture. The purpose of this article is to explain how, and under which (organisational) conditions within the requirements of the German Healthcare system, a high level of patient orientation, a more economic provision of services with digital technologies, and improved ambidexterity in leadership and organisational structure can be accomplished.

The article has been published in a German Journal (Monitor Versorgungsforschung) ^51^. Feedback showed that the topic and content should also be accessible to other disciplines for further discussions.

## Health Services 4.0 for personalised treatment pathways

Although the trends in the German healthcare system point to a higher significance of health as a benefit for the insured and for patients, healthcare in Germany is still not sufficiently oriented to the needs of the individual patient. Studies have shown that it has not been possible to meet expectations for concepts such as ‘shared decision making’ to a sufficient extent. In some disciplines, the medical profession is therefore still a long way from adopting the basic principles of professional discussion to involve the patient ^1^. This means that significant potential for the improvement of outcomes, and thus the level of effectiveness of a healthcare service, cannot be realised, and that the legal requirements in the Patient Rights Act are also not being fulfilled.

**Figure 1.**
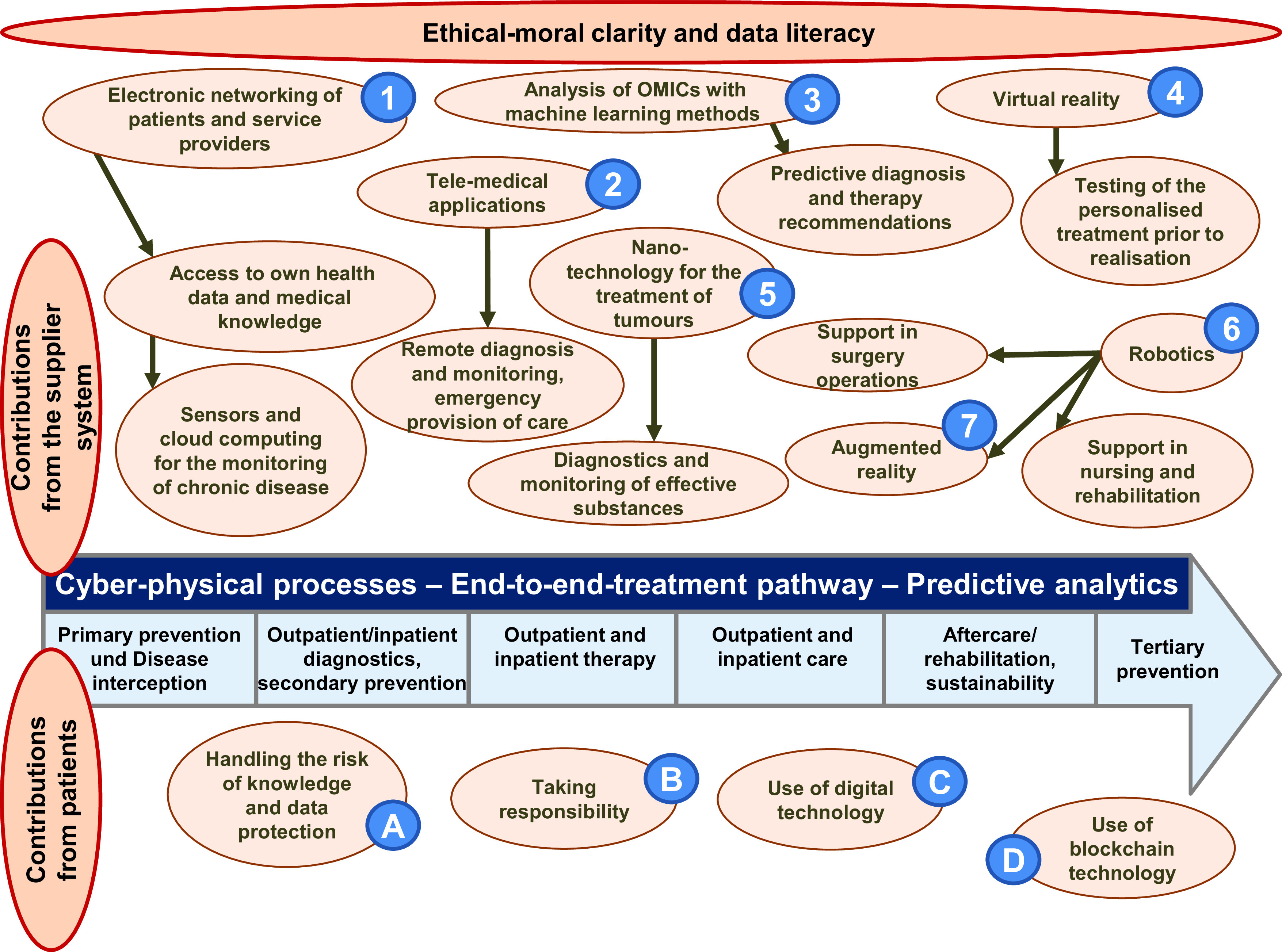
Examples for the potential of digital technologies for the personalisation of a treatment pathway ^51^.

**Figure 2.**
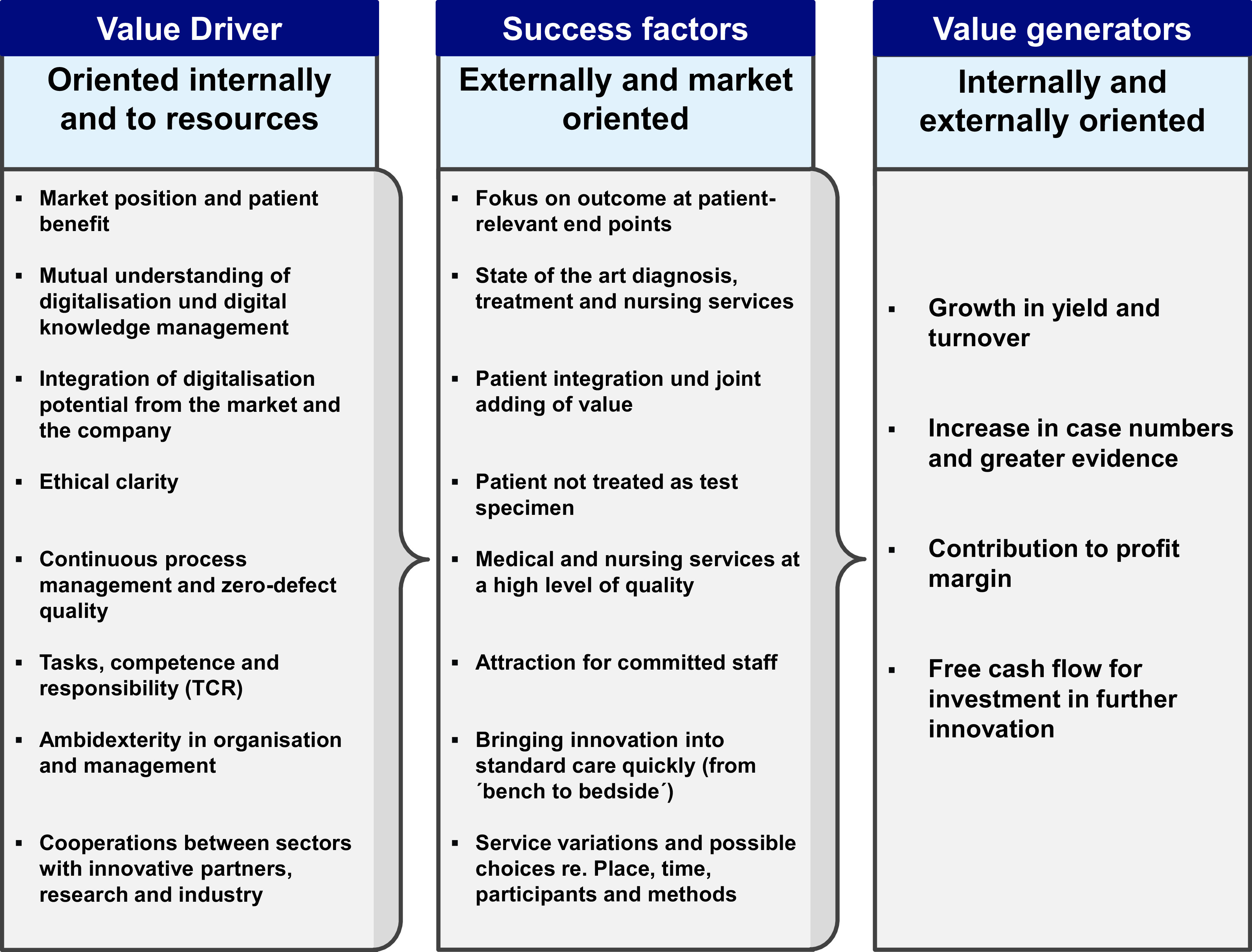
Key value drivers, success factors and value generators of a personalisation of treatment pathways ^51^.

The potential for patient integration in the form of personalised treatment pathways can be exploited through the convergence of information technology and biotechnology, so-called ‘super-convergence’ ^2^, and the resulting combination of (medical) technical innovations and their application trends. By combining suitable components of the respective technologies, Health Services 4.0 can be developed to increase the quality and value of health services (see Figure 1), and the provider can react more quickly to market developments and competition (see Figure 2).

In this way, Health Services 4.0 refers to the integration of previously standard health services, which in the German health system are financed by statutory and private health insurance funds and provided by inpatient and outpatient service providers, using digital technologies. In the resulting cyber-physical processes, central components of digitalisation are the consistent use of sensors for monitoring biomarkers, the expansion of the interaction between humans and computers, the use of mobile devices (mHealth), big-data technologies and the use of machine learning. Technologies are used here to close the gap between the virtual and the real world and are capable of interacting with people and things ^3^. In conjunction with cloud computing (i.e. where information technology resources are provided via networks), cyber-physical systems represent a cross-sectional technology for real diagnosis, therapy and care services to provide reality-based services in real time, in a virtualised and omnipresent manner with multimedia control and communication ^4^. In this way, processes that were previously analogue can be digitalised and new processes that are only available digitally – cyber-physical processes in the narrower sense – and data-driven decision support systems can be used for the real treatment of patients. They allow the linking, in compliance with data protection regulations, of standardised and personalised partial services that increase benefit in the form of Health Services 4.0. The intensive integration of the patient in this joint service creation represents a decisive lever in this process.

The *more thorough integration* of patients and relatives in the treatment process in this way means, in principle, that healthcare services are personalised in a general, conceptual approach to focus on individuals, who are comparable according to certain criteria, in order to overcome non-specific standardisation. This usually amounts to the *creation of standards for individual groups.* Here, personalisation means *even greater differentiation and detailing on specific implementation* for the individual person or the individual patient with his or her specific clinical profile and personal circumstances ^5^.

In general, *the added value of a service* is generated in the direct interaction of the provider system with the contributions of the customer (see Figure 1). Therefore, the treatment processes that create value directly represent the most important lever to achieve competitive advantages. The personalisation of treatment processes - ideally in the form of treatment pathways - thus forms the linchpin for the strategy, organisation and implementation, and for the effectiveness of treatment programmes and sequences ^6^. Consistent process management on the basis of an ethical and moral foundation with respect to the importance of a health service and 'data literacy', i.e. the ability to handle data in a competent manner ^7^, creates the prerequisites for increasing patient benefit, productivity, and outcome, and is thus the most important value driver, ensuring that focus on the patient is maintained in all diagnostic, treatment and care services.

## The potential of digital technologies for the personalisation of treatment processes in various medical fields

In general, digital technologies have considerable potential to provide higher quality and simultaneously more economical inpatient, outpatient and cross-sectoral care. This is achieved both by accelerating the communication and coordination processes between patients and service providers as well as between the service providers themselves and by developing innovative diagnosis and treatment procedures in the individual medical fields. The following are examples of technologies that can be used by healthcare providers to personalise a treatment pathway (see Figure 1).

The **electronic networking** of patients with service providers and between these providers offers many opportunities to make the service processes in prevention, diagnostics, therapy and aftercare more efficient and effective and to involve patients more closely in the treatment of chronic diseases by using apps and sensors to measure biomarkers. Through electronic networking, patients gain easier access to their own medical data and to medical knowledge and can thus become customers in the 'health market' ^8^. One example of the possibilities of networking at the system level in Germany is the 'Healthy Kinzigtal Valley' ^9^ value-added network, a cooperation of various general practitioners, specialists and hospital doctors, psychotherapists, nursing and rehabilitation facilities. Within the framework of a contract for integrated care, it plans and coordinates the treatment of insured members of certain health insurance funds. The focus of the business model here is on innovations that create new product features in conjunction with new business models and on the use of innovative information, communication and big data technologies to achieve results, i.e. concrete effects rather than an increase in the volume of services.

The provision of **tele-medical services** was approved in 2018 by an amendment of the model professional code of conduct for physicians and further developed at the 121 ^st^ Ärztetag Medical Congress in Frankfurt in 2018. This defined the relevant benefits - from the medical perspective - for the provision of telemedical services. In the broadest sense, this is understood as the overcoming of temporal and/or spatial distances in the context of medical issues. This mainly includes the measurement, recording and transmission of information or the application of medical procedures with the help of information and communication technology between doctors and between doctors and patients, if necessary with the involvement of non-physician specialists ^10^. There are now already a number of examples of applications, such as a remote application for the care and continuous aftercare of patients with cardiac insufficiency (telecoaching) or the ambulant aftercare of stroke patients (telestroke). Telemedicine services have great potential to adapt to the preferences of the patient, but there are fewer benefits for the physician providing these services. The physician must provide the time for the service both online and offline, and the set-up time for the telemedical infrastructure must be added to this.

From the analysis of gene sequences (genomics), epigenetic modifications (epigenomics), RNA transcripts (transcriptomics), proteins (proteomics) and metabolic products (metabolomics) ^11^, the so-called **OMICS technologies,** it is possible to identify biological and psycho-social subtypes of diseases that are incongruent with traditional diagnostic categories by using machine learning techniques from large amounts of data. Based on the classification, predictive therapy recommendations can be given and treatment effects can be predicted with high accuracy ^12^.

An example of the significance of genetic research and the importance of translational orientation for future targeted treatments is, for example, the discovery of 30 new genetic variants associated with depression. More effective drugs with fewer side effects can be expected for certain patient groups on the basis of further research ^13^.

Another example of advances in this area is the treatment of acute lymphatic leukaemia in children, a specific form of lymphoma that can be carried out individually for each patient using CAR-T, or the development of 'chimeric antigen receptors'. This enables leukaemia cells to be detected and killed. First results show complete remission in some cases and the absence of visible signs of tumour in the computer tomogram ^14^. Another advantage of this treatment method is that this form of cancer therapy could replace stem cell transplantation in the future ^15^.

**Virtual reality** applications, i.e. the imitation of physical reality with all its physical properties in a computer-generated, interactive, virtual environment ^16^, can be used in the training of medical students and the testing of personalised treatment approaches. For example, heart function can be modelled with a computational method, supported by artificial intelligence (digital twin) ^17^, and tailor-made cyber-physical treatments can be tested with the interdisciplinary integration of clinical patient data before the actual implementation. This technology can also be used in the treatment of anxiety disorders ^18^ and addictions ^19^.

**Nanotechnology** can be used to produce tiny devices and particles that are used in the human body for *in vivo* diagnostics and for the transport of active substances in drugs ^20^.

**Robotics** are controlled by computers with artificial intelligence programmes. Depending on the software used, they can be used, for example, as support for physically arduous work in nursing and rehabilitation, and as support systems in the operating theatre.

Researchers have used an interdisciplinary approach involving methods from neurotechnology, clinical neurology, robotics and computer simulation to develop prostheses with which patients can move more naturally. Electrical impulses are simulated so that the brain interprets them as if they came from a natural arm, enabling patients to grip more precisely ^21^. Individual operation and support processes can be structured, digitalised and automated with Brainlab. The operation steps are announced, processed and simultaneously documented. This achieves a better quality of results and greater efficiency ^22^.

The MicroSurge robot system was developed at the German Aerospace Centre (DLR) for use in minimally-invasive procedures. The devices are held by robot arms and remotely controlled by the surgeon. The system not only provides a visual insight into the operating area, but even enables haptic feedback ^23^. In combination with **Augmented Reality**, with which the user experiences the real world supplemented by a virtual component ^16^, it is possible to practise complex operations and plan them in detail or to use the method for intra-operative support. With SpectoVive, real-time computed tomography data can be converted into a three-dimensional virtual environment that provides the surgeon with a realistic representation of the environment ^24^.

The extraction of new information from all this data, which arises or is generated from the use of these technologies and may also have been collected for other purposes, is a core idea in big data ^25^. In a structured treatment pathway, these analyses can also contribute to improving treatment directly to the patient. This is done in such a way that predictive analytics ^26^ can be used in an established data warehouse to derive prognostic statements from the large quantities of information from various sources and stored in so-called big data lakes ^26^ (see Figure 1).

## Previous obstacles and barriers to digital solutions in the German healthcare system

In the German healthcare system, however, there are still considerable obstacles and barriers that make the adoption and application of innovative and digital services difficult or impossible.

Implementation is hampered by the extensive incompatibility of information and communication systems, which make it difficult to integrate data and use it across locations because of the lack of structural equality. In the medical informatics initiative DIFUTURE, universities of excellence and clinical partners have therefore joined forces to bring together the necessary data volumes from various research areas and digital products so that every doctor, researcher and patient has access to the information they need ^27^. In addition to the obstacles relating to data protection and data security, common standards are also lacking, and not all health professionals are convinced by digital techniques or trained in their use ^28^.

A further difficulty for the translation of innovations is that the costs are only covered by the health insurance funds once they have proven their clinical benefit, which is difficult given the relatively small number of cases. A change with regard to reimbursement is currently being initiated with NOVARTIS, which has agreed a reimbursement model for CART-T cell therapy using company health insurance funds. In this model, the insurance company is returned a portion of the cost of the treatment drug (Kymriah®) if the patient dies within a predefined period of time ^29^.

While hospitals are subject to the so-called proviso of prohibition regarding the use of innovative methods, the outpatient sector, in contrast, is subject to the proviso of permission ^30^. New examination and treatment methods (NUB) may be provided in the inpatient sector until the Federal Joint Committee (G-BA) has carried out an evaluation. In the outpatient sector, the proviso that permission be obtained means that services can only be provided after the Federal Joint Committee has examined, evaluated and permitted them in advance. In the current health policy discussion, it is becoming apparent that the Ministry of Health is also criticising the long decision-making processes of self-administration and is planning changes ^31^.

In general, the close and cross-sectoral cooperation of clinicians with innovative partners, namely research institutions and the medical industry, represents an important value driver for the translation of innovative services (see Figure 2). As an important success factor for patients, it offers innovative service variants and choice in terms of place, time, participants and methods at a highly medical quality and state-of-the-art research.

## Expansion of the value-added chain ‘treatment pathways’ – Dissolution of organisational divisions

In the future, healthcare will take place less in the waiting rooms of registered doctors and hospitals and more often on a smartphone ^32^. The physical dependence of many service processes on the presence of the patient can be eliminated under the conditions of digital infrastructure and interconnectivity. Sensor technology and smart diagnostic devices at the point of care provide comprehensive diagnostics independently of the respective supplier and, ideally, compatibly with the relevant systems. Telemedical service processes allow value-added chains in the form of treatment pathways to be developed further and extended, so that previously unknown or unfeasible methods and new business models can emerge. This means that there are considerably more options available to map healthcare services across all three sectors (outpatient, inpatient, home) in a continuous end-to-end treatment pathway and to provide personalised treatment (see below in Figure 1). Healthcare services can thus be personalised - oriented to the optimum course of action for the patient ^5^.

If the data from the above-mentioned technologies is used, it will be possible to better understand how diseases arise and how processes that cause illness can be detected at an early stage in the bodies of healthy people (i.e. people without symptoms of illness). In this way, earlier and earlier diagnoses and effective interventions can be used to intervene at an early stage instead of only providing treatment once the disease has already spread ^33^. Alzheimer's dementia, for example, appears to manifest itself in some forms long before the actual onset of the disease. This gives us time to prevent the disease, possibly by as long as 20 years ^34^. In another example, following investigation of the genetic risk, effective substances can be used before the onset of a related disease caused by underlying type one diabetes ^33^.

The early approach to and integration of the patient thus makes it possible to reduce the information asymmetry between doctor and patient and, with early detection, to swiftly focus on primary prevention ^35^.

Accordingly, medicine is developing from reactive medicine with a one-size-fits-all approach to proactive and anticipatory preventive medicine, which can plan treatment more precisely with combinations of therapies instead of often having to rely on trial and error as has been the case in the past. However this requires patients to reflect on their own position with regard to the **handling of their data and the risk regarding knowing and not knowing about their current health status and treatment** ( Figure 1: A), and a debate as to where disease begins and what is therefore included in the service catalogue of the health insurance companies is needed ^36^.

For healthcare providers, this means that the chain of contact with potential patients and relatives (customer journey) must begin much earlier in order to identify significant touchpoints and proactively point out personalised solutions that can be integrated into the patient's everyday life without major friction losses.

## Joint value creation with patients and relatives

One central element of successful treatment is the integration of the patient into the diagnosis and treatment process, which is also codified in the German Patient Rights Act. The aim is to achieve more knowledge and control over the disease or health service as well as greater compliance/adherence.

Integration gives the provider access to implicit patient knowledge and to feedback from which improvement processes and innovations can be developed ^37^. But patients and relatives can also act as innovators in their function as users. Their knowledge about the disease represents a great potential for process and product innovation, especially in the field of rare diseases ^38^. This requires the willingness of the patient to **take responsibility** for the recovery process and to give feedback to the practitioner ( Figure 1: B).

Smartphones and wearables, i.e. computer systems that are worn on the body and/or integrated into clothing, make patient integration possible independent of time and place. These systems can be used with digital technologies such as telemedical health services and open the possibility of using powerful sensors in conjunction with cloud computing (see Figure 1). They thus represent a considerable increase in benefit to the patient, who must be willing and able to **use the technologie**s, in the context of prevention, diagnosis and treatment ( Figure 1: C). In the context of modelling a treatment pathway on the basis of a treatment concept, such personalisation promotes the relationship between provider and patient in the sense of a specific customer orientation and creates interactive added value in the sense of a co-design process ^39^.

**Blockchain technology** ( Figure 1: D) can be used to create a system that collects all data that can only be released by the owner of the data to certain users ^40^. This would allow significant problems such as the dispersed nature and the security of medical and social data to be resolved. Blockchain data is more complete, accurate, trustworthy and widely available ^20^. It would then no longer be possible for data to be used for other purposes without the knowledge of patients. This would give the patient more security and autonomy in dealing with healthcare providers.

This development will fundamentally change the role of the doctor. By making information and knowledge accessible and easy for all to access, the physician will no longer be responsible for the transfer of knowledge as an indispensable knowledge carrier, but rather operate in the role of a consultant and coach. Doctors will instead configure a personalised preventive and/or treatment path with the patient using Health Services 4.0 and control the entire process with empathy and ethical clarity, so that the patient does not feel like a ‘test specimen’.

## Making new knowledge effective

Digitalisation will also change the future of work in the health sector, albeit not (yet) as radically as is the case for Industry 4.0 with its framework conditions of Volatility, Uncertainty, Complexity and Ambiguity (VUCA) ^41^. Even if the challenges of Google, Apple, Facebook, Amazon (GAFA) and many small health apps acting as drivers, the persistent forces in the individual sectors of the German healthcare industry still appear very strong. In this sector, which is characterised by complexity due to regulations, different incentive and billing systems, quality assurance measures, security routines and data protection regulations, the more difficult framework conditions as in Industry 4.0 do not yet apply. Nevertheless, digitalisation and the focus on translation of concepts from industry frameworks in the German healthcare system are accompanied by changes in work and organisational structures and a different management culture.

The challenges for providers are to enable the organisation and employees to develop their own innovations and to take over innovative diagnosis, treatment and care services from pharmaceutical and medical research institutions. This makes the management of automated and non-automated treatment processes and their interconnection to quality and risk management with zero-defect quality into the most important value drivers and into success-critical prerequisites for entrepreneurial action as a provider of healthcare services ( Figure 2).

The healthcare provider must constantly rebalance the conflicting goals of *exploitation* and *exploration.* New knowledge is created by specialising and refining existing knowledge in a continuous improvement process during exploitation as use and utilisation; new knowledge is developed during exploration by creating radically new application and solution possibilities in the interests of users ^42^. Both approaches can be pursued by realising ambidexterity in organisational structure and management behaviour - an approach that is also relevant for expert organisations in the healthcare sector in a time of digital transformation. This dynamic ability represents a key value driver for a health organisation to become or remain competitive in the long term.

Ambidexterity can be implemented in different organisational models depending on size, available resources and the chosen innovation strategy:

• In a cooperative organisational model, exploration is carried out in certain domains while exploitation is carried out in others (domain ambidexterity), for example in the concept of a dual-operating system ^43^.
• In the autonomous organisational model, a company carries out exploration and exploitation in separate organisational units, each of which is managed by autonomous units (structural ambidexterity).
• In the case of temporal ambidexterity, a company periodically alternates between the phases of exploration and exploitation.
• In the integrated organisational model, the business units conduct exploration and exploitation in parallel, i.e. the responsibility for innovations is with a single party (contextual ambidexterity) ^44^.

All four forms of organisation have their justifications and chances of success. In the case of Health Services 4.0, the fourth organisational model represents the greatest potential for success, alongside the third. Organisations in the German healthcare system are subject to a multitude of regulations and budgets, which usually do not allow scope for additional units, so that contextual ambidexterity in an integrated organisational model is the predominant option. Ambidexterity has also been examined closely in networks ^45^ and is therefore also relevant for transposition to physician networks, which are playing an increasingly large role in supply within the German healthcare system.

Digitalisation creates new forms of cooperation with one another, but also with machines and cyber systems, so that we are already speaking of Work 4.0 ^41^. Due to the fusion of software, hardware and new diagnostic and treatment services, established patterns of thought and routines will have to be questioned in general in order to compete for more demanding and informed patients. The focus on translational orientation with the consistent use of innovative diagnosis and treatment methods and their mapping in personalised treatment pathways requires a framework that enables innovative thinking and action, consistently processes knowledge and makes it available to everyone - including patients. It is not the knowledge itself that is in the foreground, but rather the methodological competence to apply this knowledge effectively for the individual health service ^6^. In principle, this requires a managerial, value-based attitude and an ethical, value-based attitude in the focus of several groups of stakeholders, in which patient orientation and ‘clinical credibility’ ^46^ are just as firmly anchored as a consistently business-oriented focus. This will require a corporate culture that is oriented towards both. Self-organisation and participation, development of teams away from hierarchical control and towards self-control, self-organisation to the point of independence ^47^ and to the entrepreneur within the company are key elements of design and levels of maturity ^48^.

What is needed is an approach to leadership that masters the ‘slider' of transactional and transformational leadership, that can accompany the change in the range of activities and role models as well as the increasing demands on the technical and emotional skills of employees. This adds ‘both-handedness’ of management to the organisational ambidexterity ^49^, a qualification that is directed firstly towards efficiency and excellent (the ‘left hand’) and secondly more strongly towards speed and innovation (the ‘right hand’) and can also withstand and communicate this balancing act between great openness and tight management ^50^.

## Conclusion

Digital technologies already exist in many sectors and are gradually being adopted in medicine, although - particularly in the German healthcare system - many (professional) legal, health policy and ethical issues are still completely unresolved and the debate on them can hardly keep pace with the development of digitisation technologies. There are many opportunities and justified prospects for high-quality, precise diagnosis and treatment services with fewer side effects, which can be used proactively and in a personalised manner in various business models. It remains to be seen whether the hopes for a more economical supply can be realised.

In addition to legal and technical issues, the main competitive challenges for providers are to develop process and product innovations in a continuously changing working environment and/or to incorporate and adapt technical medical and pharmacological innovations from collaborations with research and development into their own service processes.

In times of digital change, a company's ability to achieve an ambidexterity of organisation and management is a dynamic ability that provides sufficient flexibility for new developments and sufficient stability for a value- and values-oriented attitude.

## Data availability

No data are associated with this article.

## References

[1] Schmacke N : Der Schlüssel zur Partizipation. *Monitor Versorgungsforschung* 2018;3:40–42.

[2] Hahn H Schreiber A : Potenziale der Digitalen Transformation in der Medizin. In: Neugebauer R Editor. *Digitalisierung – Schlüsseltechnologien für Wirtschaft & Gesellschaft* Berlin Heidelberg: Fraunhofer Forschungsfokus;2018. p.323–345.

[3] Bruhn M Hadwich K : Relevanz von Dienstleistungen 4.0 in Wissenschaft und Praxis. In: Bruhn M Hadwich K Editor. *Dienstleistungen 4.0 – Konzepte – Methoden – Instrumente. Band I. Wiesbaden* 2017. p.5–40.

[4] acatech: Integrierte Forschungsagenda Cyber-Physical System. *München* 2012.

[5] Töpfer A Brabänder G : Personalisierung von Gesundheitsleistungen – Gestaltungsempfehlungen zur Umsetzung. *ZEFQ* 2018;130:27–34. 10.1016/j.zefq.2017.12.002 29329961

[6] Stöger R : Prozessmanagement. In: 4 ^th^ Edition *Stuttgart* 2018; p.IX.

[7] Kuhn S : Transformation durch Bildung. *Deutsches Ärzteblatt* Volume2018;115,14:A633–A638.

[8] Wilhelm A : Der Patient im deutschen Gesundheitssystem: Einstellungen, Präferenzen und Erwartungen. In: Gellner W Wilhelm A Editor. *Vom klassischen Patienten zum Entrepreneur.* Baden Baden;2006, p.18–57.

[9] Hildebrandt H : Sprunginnovationen in der Organisation der Versorgung. *Versorgungsforschung* 2014;7:29–33. 10.24945/MVF.01.14.1866-0533.1929

[10] Kassenärztliche Bundesvereinigung: Telemedizin. Reference SourceLast accessed on 18.03.2019.

[11] Leopoldina –Nationale Akademie der Wissenschaften: Zukunftsreport Wissenschaft. Reference SourceLast accessed on 14.03.2019.

[12] Bzdok D Karrer TM Habel U : Big-Data-Ansätze in der Psychiatrie: Beispiele aus der Depressionsforschung. *Der Nervenarzt* 2018;89:869–874. 29188348 10.1007/s00115-017-0456-2

[13] Wray NR Ripke S Mattheisen M : Genome-wide association analyses identify 44 risk variants and refine the genetic architecture of major depression. Reference SourceLast accessed on 21.03.2019. 10.1038/s41588-018-0090-3 PMC5934326 29700475

[14] Chustecka Z : Leukämie und Lymphome: Euphorie über CAR-modifizierte T-Zellen als neue Therapieoption. Reference SourceLast accessed on 21.03.2019.

[15] Reisinger K : Zielgerichtetes Instrument. Reference SourceLast accessed on 21.03.2019.

[16] Specht P : Die 50 wichtigsten Themen der Digitalisierung, München. 2018.

[17] Wallenfels W : Digitale Zwillinge – ein Gipfelhighlight. Reference SourceLast accessed on 21.03.2019.

[18] Ärztezeitung: Heilen mit künstlicher Wirklichkeit. Reference SourceLast accessed on 14.03.2019.

[19] Ärztezeitung: VR-Brille statt Zigarette. Reference SourceLast accessed on 21.03.2019.

[20] Bogdan B : *MedRevolution – Neue Technologien am Puls der Patienten* Berlin 2018.

[21] Ärztezeitung: Armprothesen mit Feingefühl. Reference SourceLast accessed on 21.03.2019.

[22] Brainlab: Reference SourceLast accessed on 21.03.2019.

[23] DLR – Institut für Robotik und Mechatronik: Reference SourceLast accessed on 21.03.2019.

[24] University of Basel: Virtual Reality in Medicine: New Opportunities for Diagnostics and Surgical Planning. Reference SourceLast accessed on 21.03.2019.

[25] Landrock H Gadatsch A : Big Data im Gesundheitswesen kompakt. *Wiesbaden* 2018.

[26] Seiter M : Business Analytics. *München* 2017.

[27] Medizininformatik Initiative Difuture: Reference SourceLast accessed on 21.03.2019.

[28] Reference SourceLast accessed on 21.03.2019.

[29] Wallenfels M : Künstliche Intelligenz – bald Rückgrat des Versorgungsalltags? Reference SourceLast accessed on 12.03.2019.

[30] Gemeinsamer Bundesausschuss: Methodenbewertung. Reference SourceLast accessed on 21.03.2019.

[31] Frankfurter Allgemeine Zeitung: Spahn macht Fettabsaugen zur Kassenleistung. 11.01.19, p.17.

[32] Rytina S : Hauptstadt-Kongress erörtert Künstliche Intelligenz: “Das Gesundheitssystem der Zukunft findet auf dem Smartphone statt.„ Reference SourceLast accessed on 24.11.2020.

[33] Hofmann S Telgheder M : “Wir erleben eine neue Ära der Medizin.„ Reference SourceLast accessed on 21.03.2019.

[34] Müller T : Alzeimer-Anzeichen schon 25 Jahre vor Ausbruch. Reference SourceLast accessed on 24.11.2010.

[35] Arbeitskreis Ökonomie im Gesundheitswesen der Schmalen-Gesellschaft für Betriebswirtschaft e.V. Digitalisierung im Krankenhaus: Technische Entwicklungen und deren Implikationen für Behandlungsprozesse. In: Krause S Pellens B . Editor. Wiesbaden: Betriebswirtschaftliche Implikationen der digitalen Transformation;2018, p.203–219.

[36] Streek H : Das sagen Experten zu dem neuen Forschungsgebiet. Reference SourceLast accessed on 24.11.2020.

[37] Lucius-Hoene G Palant A : Zwei verschiedene Welten – Wie medizinisches Personal von Patientenerfahrungen lernen kann. *KU Gesundheitsmanagement* 2015;84:69–71.

[38] Habicht H Oliveira P Shcherbatiuk V : User Innovators: When Patients Set Out to Help Themselves and End Up Helping Many. *Die Unternehmung* 2013;66(3):277–294.

[39] Reichwald R Piller F.T : Interaktive Wertschöpfung. In: 2 ^nd^ Edition *Wiesbaden* 2009.

[40] Perez S : Proof Work aims to decentralize medical data by using the blockchain. Reference SourceLast accessed on 24.11.2020.

[41] Bruckner L Werther S : Allgemeiner Überblick über Arbeit 4.0. In: Werther S Bruckner L Editor. *Arbeit 4.0 aktiv gestalten* Berlin 2018, p.15–21.

[42] March JG : Exploration and Exploitation in organizational Learning. *Org Sci* 1991;2(1), p.71–87.

[43] Kotter JB : Accelerate. In: *München* 2015.

[44] Ebers M : Organisationsmodelle für Innovation. *ZfbF* 2017;69:81–109.

[45] Wessel L Gersch M Goeke C : Netzwerk-Ambidextrie: Ist eine Balance explorativen und exploitativen Lernens auch in Netzwerken möglich?In: Stephan M Kerber W Editor. *Jahrbuch Strategisches Kompetenz-Management, Volume 4: „Ambidextrie“: Der unternehmerische Drahtseilakt zwischen Ressourcenexploration und –exploitation* München Mering 2010, p.121–147.

[46] Koch T Töpfer A Heller A.R : Team Management im Krankenhaus für eine Lernkultur. In: Albrecht DM Töpfer A (Editor). *Handbuch Changemanagement im Krankenhaus* , 2 ^nd^ Edition Berlin 2017, p.587–608.

[47] Kaltenecker S : Selbstorganisierte Unternehmen – Management und Coaching in der agilen Welt. *Heidelberg* 2017.

[48] Töpfer A Duchmann C : Motivationseffekte bei den Mitarbeitern durch die Krankenhaus-Kultur. In: Albrecht DM Töpfer A Editor. *Handbuch Changemanagement im Krankenhaus* 2 ^nd^ Edition Berlin 2017, p.529–537.

[49] Burkhart S Grabmeier S : Der Einfluss von Digital Leadership auf Organisationen im Gesundheitswesen. In: Matusiewicz D Pittelkau C Elmer A Editor. *Die Digitale Transformation im Gesundheitswesen* Berlin 2017, p.255–261.

[50] Duwe J : Beidhändige Führung. *Stuttgart* 2018.

[51] Töpfer A Brabänder G : Personalisierung von Behandlungspfaden – Das Potenzial digitaler Technologien. *Monitor Versorgungsforschung* 2019;7(05).

