# Supplementary material for: Personalisation of treatment pathways – analysis of chances and barriers by the implementation of digital technologies under the conditions of the German Health System
Source: F1000Res. 2021 Feb 25;10:147. [Version 1] doi: 10.12688/f1000research.29969.1 (PMC8515494; doi:10.12688/f1000research.29969.1)
Supplement: Peer review history

# Peer review: Reviewer response for version 1

**DOI:** 10.5256/f1000research.33017.r95623

**Published:** 2021-10-13

**Authors:** Maurice Mars

**Affiliations:** 1 Department of TeleHealth, Nelson R. Mandela School of Medicine, University of KwaZulu-Natal, Durban, South Africa

Is the topic of the opinion article discussed accurately in the context of the current literature?

 The issue of providing personalised healthcare through the judicious use of digital technologies is of global interest. The paper is from the German perspective of its legislative, funding, health system and service provision milieu. Germany is in the interesting position of having a Patients’ Rights Act, promulgated in 2013, a Digital Health Act passed in 2019 and being a late adopter of aspects of telemedicine in 2018. As the paper is a translation of a 2019 paper in German it would be interesting to know whether subsequent developments, especially the Digital Health Act and technology advances have altered any of the points made.

 It would be helpful for the average reader to have the concept of ambidexterity explained in the introductory section.

 Are all factual statements correct and adequately supported by citations?

 Most of the cited papers are in German and so have not been checked. There are no obvious errors in the information provided.  

 Are arguments sufficiently supported by evidence from the published literature?

 See the comment above.

 Are the conclusions drawn balanced and justified on the basis of the presented arguments?

 The matter of the 'completely unresolved' legal, health policy and ethical issues raised in the conclusion is not addressed in the body of the paper.

Is the topic of the opinion article discussed accurately in the context of the current literature?

Yes

Are arguments sufficiently supported by evidence from the published literature?

Yes

Are all factual statements correct and adequately supported by citations?

Partly

Are the conclusions drawn balanced and justified on the basis of the presented arguments?

Partly

Reviewer Expertise:

Telemedicine and eHealth, implementation, and related legal and ethical issues.

I confirm that I have read this submission and believe that I have an appropriate level of expertise to confirm that it is of an acceptable scientific standard.

# Peer review: Reviewer response for version 1

**DOI:** 10.5256/f1000research.33017.r80263

**Published:** 2021-03-12

**Authors:** Aline Gottlieb

**Affiliations:** 1 Department of Physiology, Johns Hopkins School of Medicine, Baltimore, MD, USA

Is the topic discussed accurately? In context of current literature?

- The topic is very accurately discussed from a German perspective. The provided literature sources are accurate and deliver a good overview from older citations (to depict the beginning of developments) to recent sources and updates.
- One point to mention (which is based on the nature of what the paper is describing): almost all cited sources are in German. This makes it more difficult for an international audience to follow up/ go deeper in the topic. I would advise the authors to try to provide more English sources where possible.

Are all factual statements correct and adequately supported by citations?

 A couple of times, statements need to be supported with additional citations or need clarification, for example:

- p 3: sources for funding “health systems are financed by statutory and private health insurance funds and provided by inpatient and outpatient service providers, using digital technologies”
- same page: “more thorough interpretation: is written in italic and it is unclear to me why ( is this a term introduced by the authors? – needs more clarification)

I would recommend that the authors critically look at especially sections on page 3 and 4 to ensure proper citing of all statements that are not their own.

 Are arguments sufficiently supported by evidence from published literature?

- Especially the section in the paper highlighting ongoing examples of how developments in technology and medicine can be linked, are very well cited and explained for a broader audience.

Are the conclusions drawn balanced and justified on the basis of the presented arguments?

- Overall, drawn conclusions are clear and well-presented from the economic point of view. As a suggestion, I would recommend trying to link more clearly how ambidextry influences/affects the intertwining of digitalization and medical knowledge (and patient diagnostics and treatment)

Is the topic of the opinion article discussed accurately in the context of the current literature?

Yes

Are arguments sufficiently supported by evidence from the published literature?

Yes

Are all factual statements correct and adequately supported by citations?

Partly

Are the conclusions drawn balanced and justified on the basis of the presented arguments?

Partly

Reviewer Expertise:

gastroenterology, hepatology, digitization processes and developments in  health/care, medicine

I confirm that I have read this submission and believe that I have an appropriate level of expertise to confirm that it is of an acceptable scientific standard.
